# Robust Object Detection in Adverse Weather Conditions: ECL-YOLOv11 for Automotive Vision Systems

**DOI:** 10.3390/s26010304

**Published:** 2026-01-02

**Authors:** Zhaohui Liu, Jiaxu Zhang, Xiaojun Zhang, Hongle Song

**Affiliations:** College of Transportation, Shandong University of Science and Technology, Qingdao 266590, China; 13956475095@163.com (J.Z.); pnxxzxj@163.com (X.Z.); honglest@163.com (H.S.)

**Keywords:** adverse weather, automotive vision, edge-enhancement, multi-scale fusion, lightweight detection

## Abstract

The rapid development of intelligent transportation systems and autonomous driving technologies has made visual perception a key component in ensuring safety and improving efficiency in complex traffic environments. As a core task in visual perception, object detection directly affects the reliability of downstream modules such as path planning and decision control. However, adverse weather conditions (e.g., fog, rain, and snow) significantly degrade image quality—causing texture blurring, reduced contrast, and increased noise—which in turn weakens the robustness of traditional detection models and raises potential traffic safety risks. To address this challenge, this paper proposes an enhanced object detection framework, ECL-YOLOv11 (Edge-enhanced, Context-guided, and Lightweight YOLOv11), designed to improve detection accuracy and real-time performance under adverse weather conditions, thereby providing a reliable solution for in-vehicle perception systems. The ECL-YOLOv11 architecture integrates three key modules: (1) a Convolutional Edge-enhancement (CE) module that fuses edge features extracted by Sobel operators with convolutional features to explicitly retain boundary and contour information, thereby alleviating feature degradation and improving localization accuracy under low-visibility conditions; (2) a Context-guided Multi-scale Fusion Network (AENet) that enhances perception of small and distant objects through multi-scale feature integration and context modeling, improving semantic consistency and detection stability in complex scenes; and (3) a Lightweight Shared Convolutional Detection Head (LDHead) that adopts shared convolutions and GroupNorm normalization to optimize computational efficiency, reduce inference latency, and satisfy the real-time requirements of on-board systems. Experimental results show that ECL-YOLOv11 achieves mAP@50 and mAP@50–95 values of 62.7% and 40.5%, respectively, representing improvements of 1.3% and 0.8% over the baseline YOLOv11, while the Precision reaches 73.1%. The model achieves a balanced trade-off between accuracy and inference speed, operating at 237.8 FPS on standard hardware. Ablation studies confirm the independent effectiveness of each proposed module in feature enhancement, multi-scale fusion, and lightweight detection, while their integration further improves overall performance. Qualitative visualizations demonstrate that ECL-YOLOv11 maintains high-confidence detections across varying motion states and adverse weather conditions, avoiding category confusion and missed detections. These results indicate that the proposed framework provides a reliable and adaptable foundation for all-weather perception in autonomous driving systems, ensuring both operational safety and real-time responsiveness.

## 1. Introduction

With the rapid development of intelligent transportation systems and autonomous driving technologies, visual perception has become one of the core technologies for ensuring safety and improving efficiency in complex traffic environments. Object detection, as a key task in visual perception, directly impacts the reliability of downstream functions such as path planning and decision control [1,2]. However, in practical applications, adverse weather conditions (such as fog, rain, and snow) remain a significant challenge for object detection. These weather factors significantly degrade image quality, causing blurriness, reduced contrast, and increased noise, which severely weakens the robustness of traditional object detection frameworks [3,4]. In low-visibility environments, stable detection of vehicles and pedestrians is particularly crucial, as detection failures can directly lead to traffic safety incidents [5].

In recent years, object detection methods based on convolutional neural networks (CNNs)—such as Faster R-CNN [6], the YOLO series [7], and SSD [8], have achieved remarkable progress in conventional scenarios, attaining a relatively balanced trade-off between detection accuracy and real-time performance. However, when visibility decreases significantly, these models often find it difficult to effectively preserve edge and texture features, resulting in blurred object boundaries, missed small targets, and category confusion. In addition, the stringent real-time requirements of automotive platforms mean that complex or high-parameter detection models can easily cause inference latency issues. Therefore, improving the robustness of detection models under adverse weather conditions while maintaining high inference efficiency has become a key challenge for intelligent driving vision systems.

To address these challenges, this study proposes an enhanced object detection framework—ECL-YOLOv11 (Edge-enhanced Context-guided Lightweight YOLOv11), building upon our previous research efforts [9,10,11], aiming to solve the robustness and real-time challenges of object detection under adverse weather conditions. Compared to the traditional YOLOv11 [12], ECL-YOLOv11 introduces three structural improvements: the Edge-Enhancement Convolution (CE) module integrated is added to the Backbone to preserve edge and contour information in images; in the Neck, the Context-Guided Multi-Scale Fusion Network (AENet) is designed to enhance the model’s capability of perceiving multi-scale features through context guidance; and the Lightweight Shared Convolution Detection Head (LDHead) in the Head, which reduces redundant computations and improves real-time inference efficiency. These three modules are structurally complementary, corresponding respectively to edge preservation, context fusion, and computational optimization, forming an integrated architecture for object detection under adverse weather conditions.

The main innovations of this paper include:

(1) For the first time, the YOLOv11 framework introduces an explicit edge enhancement mechanism, in which the CE module strengthens the edges and texture features of low-quality images.

(2) The AENet structure based on rectangular calibration and cross-scale fusion is proposed, which effectively improves the model’s small-object detection performance under low-visibility conditions.

(3) The LDHead adopts a lightweight shared design for the detection head, reducing GFLOPs while maintaining high-precision output.

(4) Under various adverse weather conditions, the proposed model demonstrates a good balance between robustness and real-time performance.

Comprehensive experimental results from multiple perspectives demonstrate the superiority of the ECL-YOLOv11 model over the YOLOv11 baseline in terms of core metrics such as mAP and Precision. Notably, it achieves this while maintaining a high inference speed of 230.3 FPS, underscoring its high usability for practical automotive vision systems. Furthermore, the model’s stable performance under various weather conditions and complex scenarios involving different motion states validates its robust generalization capability and considerable potential for deployment on vehicle terminals.

## 2. Related Work

### 2.1. Traditional CNN-Based Object Detection Methods

Over the past decade, convolutional neural networks (CNNs) have driven rapid advancements in object detection. Representative approaches include the region-proposal-based Faster R-CNN, the single-stage SSD, and the efficient YOLO family. These frameworks achieve an excellent balance between detection accuracy and real-time performance under clear weather or ideal illumination. However, in adverse weather conditions such as fog, rain, or snow, reduced image contrast and increased noise degrade edge and texture representations during high-level feature extraction, resulting in blurred object boundaries and missed small targets. In addition, anchor-based detectors experience a notable drop in localization accuracy when matching anchor boxes with ground-truth objects in low-contrast environments [13,14]. Although anchor-free models have recently improved localization precision to some extent, they still face challenges in maintaining edge preservation while ensuring fast inference under complex weather conditions.

### 2.2. Transformer-Based Detection Models

With the rapid rise of visual Transformers, object detection frameworks have increasingly shifted toward attention-based architectures. Representative models such as RT-DETR [15] and DINO [16] can model global dependencies within large receptive fields, thereby achieving stronger semantic consistency in complex backgrounds.Despite their superior performance in multi-class detection scenarios, Transformer-based models are characterized by enormous computational demands and high training costs, which pose significant challenges for real-time inference in vehicle-mounted systems, leading to unacceptable latency. Moreover, under severe contrast degradation or edge blurring, these models tend to over-rely on global context, which reduces their ability to capture local structural cues such as vehicle contours and pedestrian boundaries.

### 2.3. Detection and Enhancement Methods for Adverse Weather Conditions

Research on object detection under adverse weather generally falls into two major categories: (1) Image enhancement or dehazing-based preprocessing approaches, and (2) architectural improvements to detection models. The former, represented by TransWeather [17], MSNet [18], and other physics-based enhancement algorithms, can improve image visibility but inevitably increase computational overhead. The latter, including DAGL-Faster R-CNN [19], PC-YOLO [20], and YOLOv8-ASF [21], enhances robustness through domain adaptation, feature decoupling, or multi-scale fusion mechanisms. However, these models typically require more complex feature pyramids or multi-branch inference structures, which compromise real-time performance. Therefore, how to enhance edge preservation and contextual feature fusion while maintaining high inference efficiency remains a critical challenge in automotive object detection.

### 2.4. Contribution of This Paper

Overall, existing studies reveal a clear trade-off between robustness and real-time performance. Transformer-based architectures, though strong in semantic understanding, are computationally expensive, whereas lightweight CNN-based detectors, while efficient, suffer from feature degradation under adverse conditions. To address these limitations, this work proposes ECL-YOLOv11 (Edge-enhanced, Context-guided, and Lightweight YOLOv11), which achieves a unified balance between robustness and efficiency through three complementary modules: the CE module for edge enhancement, the AENet module for context-guided multi-scale feature fusion, and the LDHead module for lightweight real-time detection. By maintaining low computational complexity while significantly improving detection stability under adverse weather, ECL-YOLOv11 effectively bridges the gap between “efficient yet fragile” and “robust yet slow” detection frameworks.

## 3. Method

### 3.1. Edge Enhancement Convolution Module (CE)

**Figure 1:**
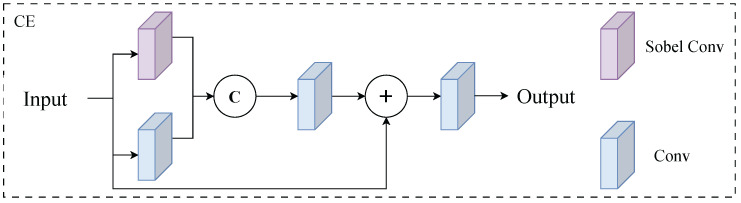
CE module structure diagram.

In adverse weather conditions, image contrast and clarity often significantly decrease. The degradation of edge and texture information makes it difficult to accurately identify object boundaries, thereby affecting detection accuracy and stability. Traditional convolutional neural networks typically weaken responses to local gradients and edge features during high-level feature extraction, a drawback particularly prominent in low-visibility environments such as fog, rain, and snow. To enhance the model’s boundary perception capability in degraded images, this paper proposes a Convolutional Edge-Enhancement Module (CE) as shown in Figure 1. The module introduces a fixed Sobel operator to extract horizontal and vertical gradients, which are then fused with learnable convolutional features. This explicitly retains edge information in the network’s feature layer, achieving dual representation of texture and semantic features.

Figure 1 illustrates the architecture of the CE module, comprising two parallel branches: the Sobel edge extraction branch and the learnable convolutional branch. The former employs fixed convolution kernels to extract image gradient features, while the latter captures contextual semantic information through 3 × 3 learnable convolutions. These features are concatenated across channels and then compressed and fused via 1 × 1 convolutions. This fusion mechanism ensures the model maintains high sensitivity to target boundaries even with low-quality inputs, effectively reducing false positives and missed detections caused by blurred edges.

**Figure 2:**
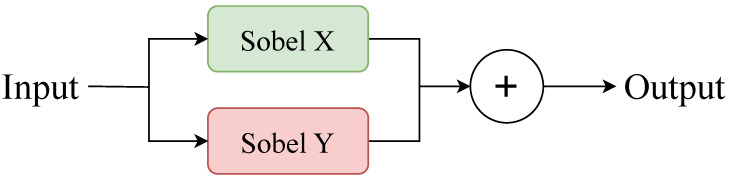
Sobel Conv framework diagram.

Figure 2 presents the architecture of the Sobel Conv framework, and its corresponding mathematical formulation is provided in Equations (1)–(3): Let the input feature be $x\in R^{C\times H\times W}$, where the Sobel kernels $K_{x}$ and $K_{y}$ are applied for horizontal and vertical gradient computation, respectively.

(1) $$K_{x}=\left[\begin{array}{ccc} 1 & 0 & -1\\ 2 & 0 & -2\\ 1 & 0 & -1 \end{array}\right],\;K_{y}=\left[\begin{array}{ccc} 1 & 2 & 1\\ 0 & 0 & 0\\ -1 & -2 & -1 \end{array}\right]$$

Edge features are derived by summing the horizontal and vertical responses as follows:

(2) $$x_{sobel}=Conv\left(x;K_{x}\right)+Conv\left(x;K_{y}\right)$$

where Conv(·;K) denotes the convolution operation with a fixed kernel. Simultaneously, the convolution branch utilizes a learnable 3×3 kernel to extract regular semantic features, as represented by:

(3) $$x_{conv}=\sigma \left(W*x+b\right)$$

Here, *W* and *b* are learnable parameters, ∗ indicates the convolution operation, and σ(·) is the nonlinear activation function. The outputs of both branches are concatenated along the channel dimension, yielding:

(4) $$x_{cat}=\left[x_{sobel},x_{conv}\right]$$

Feature compression and fusion are performed using a 1×1 convolution, which can be expressed as:

(5) $$x_{f}=\phi \left(W_{1}*x_{cat}\right)$$

where ϕ(·) denotes a nonlinear transformation, and $W_{1}$ is the learnable 1×1 convolutional parameter. To maintain the integrity of the input features and improve feature transmission efficiency, a residual connection is introduced by adding the fused features to the original input, as follows:

(6) $$x_{r}=x_{f}+x$$

Finally, the output is generated through another 1×1 convolution:

(7) $$y=W_{2}*x_{r}$$

This design combines the interpretability of fixed operators with the expressive power of learnable convolution, enabling the model to extract high-level semantics while retaining key structural edge information.

**Figure 3:**
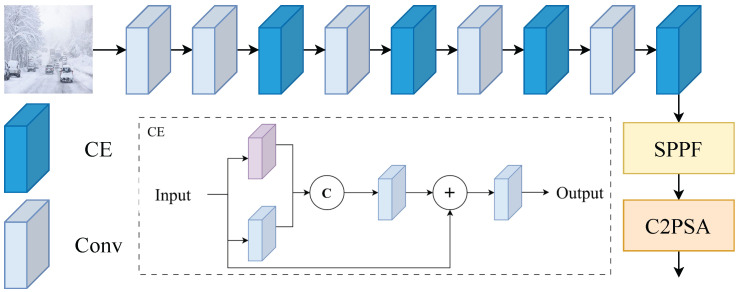
CEB framework diagram.

Furthermore, to enhance the network’s overall perception capability, this paper embeds the CE module into the YOLOv11 backbone to construct a CE-Enhanced Backbone (CEB) framework, as shown in Figure 3. This architecture inserts a lightweight edge enhancement unit into the original feature extraction path, enabling the fusion learning of low-level gradient features and high-level semantic features. Through this integration approach, the proposed model not only learns clearer target boundary features but also maintains stable detection performance under complex weather conditions. Compared with traditional convolutional blocks, this module effectively reduces boundary drift issues in blurry and low-contrast images while improving detection accuracy and robustness. Additionally, the CE module demonstrates excellent versatility, allowing flexible integration into other lightweight backbone networks to provide scalable edge-enhancement solutions for various visual tasks.

### 3.2. Context-Guided Multi-Scale Fusion Network (AENet)

In complex weather conditions, traditional object detection networks often struggle to simultaneously achieve effective performance across targets of varying scales. Particularly in low-visibility scenarios such as fog, rain, or snow, small and distant targets are prone to false positives or missed detections due to insufficient contextual information. Existing feature pyramid architectures (e.g., FPN or PAN) primarily rely on unidirectional feature propagation (top-down or bottom-up) during multi-scale fusion, which demonstrates limitations when processing images with blurred edges and low contrast. To address this, we propose a Context-Guided Multi-Scale Fusion Network (AENet). By integrating multi-level contextual fusion, rectangular auto-calibration, and bidirectional semantic flow mechanisms, the network achieves robust perception and feature enhancement for targets of different scales.

AENet replaces the Neck component of the original YOLOv11, serving as a critical feature fusion network that connects the Backbone with the detection head. Its architecture comprises four functional sub-modules: Pyramid Context Extraction (PCE), Rectangular Calibration (RCM), Down-to-Up Information Flow (DIF), and Feedback Block (FBM). These modules work through hierarchical complementarity and contextual coupling mechanisms, enabling the model to simultaneously enhance global semantic understanding and local structural details, thereby achieving stable detection performance under adverse weather conditions.

**Figure 4:**
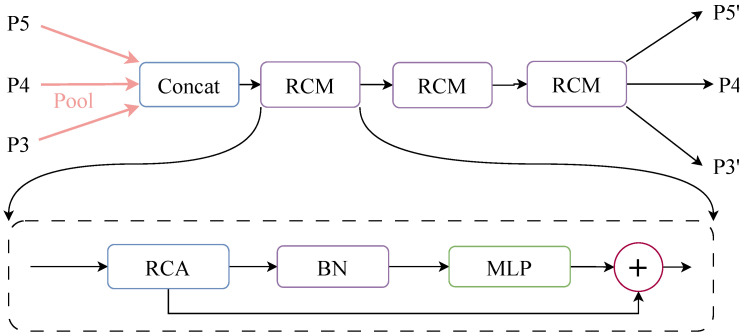
PCE module mechanism.

To unify the spatial dimensions of multi-level feature maps and extract contextual information across different scales, AENet introduces a Pyramid Context Extraction (PCE) module in its Neck section. As shown in Figure 4, this module first performs pooling on multi-scale features (e.g., $P_{3},P_{4},P_{5}$) to ensure consistent spatial dimensions. The features are then concatenated along the channel dimension and fed into multiple cascaded Rectangular Calibration Modules (RCM) to achieve hierarchical contextual enhancement. Unlike traditional convolutions, the RCM adopts a strip-convolution architecture to model long-range dependencies along both the horizontal and vertical directions, thereby capturing the elongated edge structures of rectangular targets. This design is particularly effective for detecting regular-shaped objects—such as vehicles, lanes, and pedestrians—and markedly improves structural perception in low-visibility scenes with blurred boundaries. The process is mathematically expressed in Equation (8). Finally, the concatenated feature map is split back into the corresponding feature maps for each input scale. The mathematical expression for this process is shown in Equation (9).

(8) $$Output=\sigma \;\left(Excite(AdaptiveAvgPool(x))\right)\times {\mathrm{dwconv}}_{hw}\left(x\right)$$

(9) $$x^{\prime }=RCM\left(PyramidPoolAggPCE(P_{3},P_{4},P_{5})\right)$$

Excite refers to the stripe convolution operation, σ denotes the Sigmoid activation function, and ${\mathrm{dwconv}}_{hw}$ indicates the depthwise separable convolution operation. PyramidPoolAggPCE represents the adaptive pooling and concatenation process, RCM further performs contextual enhancement along the rectangular directions (horizontal and vertical), compensating for semantic bias across multi-scale features.

**Figure 5:**
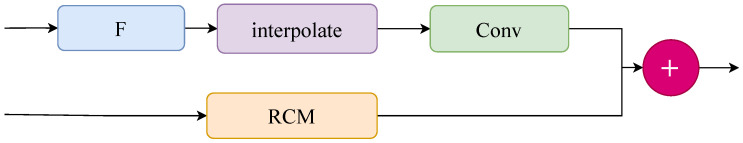
DIF module.

To further realize multi-layer semantic complementarity, AENet introduces a top-down information flow module (DIF) that transfers semantic guidance from high-level features to lower-level features. Figure 5 illustrates the DIF module’s architecture: high-level features F are first upsampled (interpolated) and channel-matched via convolutions to align with the resolution of lower-level features. Meanwhile, the lower-level features are enhanced by RCM to strengthen semantic expression. The two streams are then fused spatially to produce the output, as detailed in Equation (10).

(10) $$Out_{DIF}=x_{1}+Conv\;\left(Interp(x_{2})\right)$$

This module not only preserves the downward transmission of high-level semantic information, but also enhances the semantic representation of low-level features via RCM, effectively mitigating the problem of insufficient semantics in traditional PAN structures.

**Figure 6:**
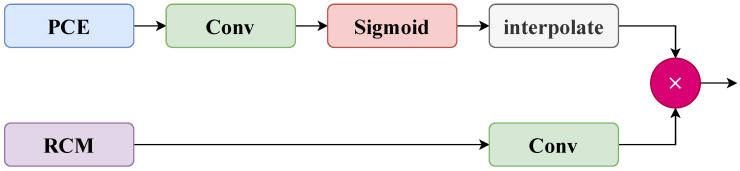
FBM module.

Unlike the DIF module, AENet’s bottom-up feedback module (FBM) facilitates detail feedback and structural reinforcement. As shown in Figure 6, this module generates a gated weight map from the high-resolution features produced by PCE and performs structure-aware fusion with low-resolution features to achieve detail compensation. The detailed process is described in Equation (11):

(11) $$Out_{FBM}=Conv\left(x_{l}\right)\times \sigma \;\left(Interp\;\left(Conv(x_{h})\right)\right)$$

where $x_{1}$ and $x_{2}$ represent the features from the lower and higher layers, respectively. $Interp(x_{2})$ refers to the upsampling operation applied to $x_{2}$. The operations $Conv(x_{l})$ and $Conv(x_{h})$ perform convolution for the low-resolution and high-resolution features, respectively, while Interp denotes the upsampling operation applied to high-resolution features.

Finally, all fused features are input into the C3K2 module in YOLOv11 for unified processing. This module employs an alternating stacked 3×3 convolutional structure to compress and refine the multi-scale fused semantic information, thereby improving the feature representation quality of the final detection head while ensuring efficient computational performance.

**Figure 7:**
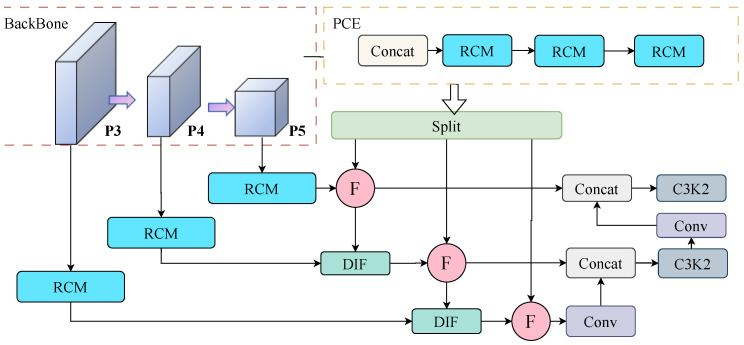
AENet network framework diagram.

In summary, the AENet architecture establishes a multi-scale fusion framework that jointly ensures semantic integrity and structural detail fidelity through a collaborative mechanism integrating Pyramid Context Extraction (PCE), Rectangular Calibration Module (RCM), Down-to-Up Information Flow (DIF), and Feedback Block Module (FBM). As shown in Figure 7, this framework sustains robust detection across targets of different scales in complex scenes and, under adverse weather conditions, effectively enhances the model’s generalization ability and structural perception.

### 3.3. Lightweight Shared Convolution Detection Head (LDHead)

**Figure 8:**
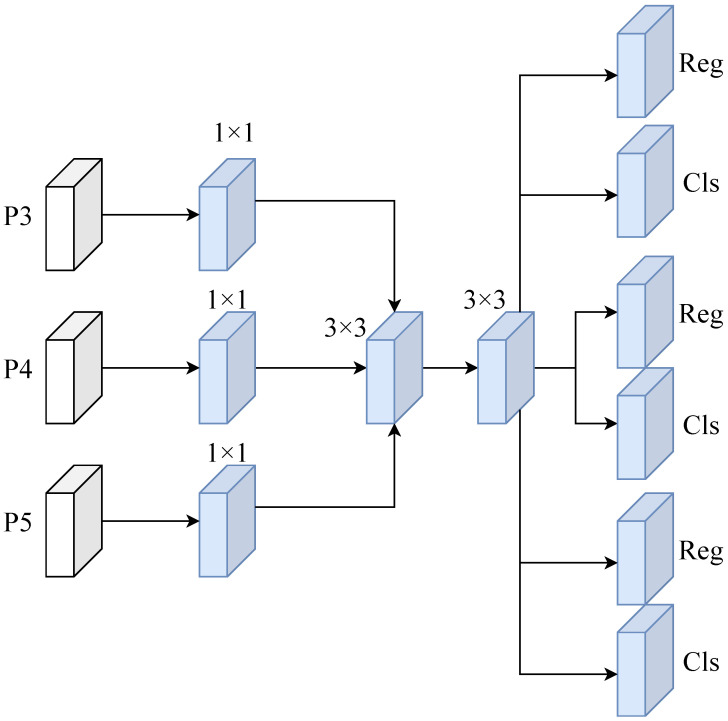
LDHead framework diagram.

Autonomous driving vehicle systems require extremely high real-time performance from object detection models. Overly complex detection head designs often significantly increase computational load, leading to inference delays and degraded deployment performance. To reduce computational costs while maintaining detection accuracy, this paper proposes a lightweight shared convolutional detection head (LDHead) architecture for efficient and stable object detection on automotive platforms. Traditional detection heads independently construct convolutional branches on each scale’s feature maps, which enhances feature representation at each layer but results in excessive parameter redundancy and computational overhead. The core concept of LDHead simplifies the architecture through “cross-scale parameter sharing”: sharing a unified set of convolutional weights across multi-scale feature maps, thereby maintaining representational power while substantially reducing computational complexity. Additionally, LDHead employs depthwise separable convolution and GroupNorm normalization strategies to achieve efficient feature modeling and stable distribution alignment. This module replaces the original multi-head detection layer in the YOLOv11 architecture, uniformly processing multi-scale features (P3, P4, P5) from the AENet output to form the final detection prediction stage, as shown in Figure 8.

As shown in Figure 8, the LDHead module takes multi-scale features from AENet’s output as input. Each feature map undergoes standardization through a 1×1 convolution and GroupNorm, reducing statistical discrepancies across scales. The processed multi-scale features are then passed into a shared convolutional structure composed of 3×3 depthwise separable convolutions, which extract features in both spatial and channel dimensions. This shared-parameter mechanism enables the network to obtain unified feature representations across multiple scales while significantly reducing redundant convolutional operations. The shared convolution outputs are split into two independent branches: one for bounding box regression and the other for class prediction. The regression branch outputs 4×reg_max channels through 1×1 convolution, representing the bounding box offsets in four directions, and is further processed by the regression module for size adjustment. The classification branch outputs the class probability distribution through 1×1 convolution and passes through a sigmoid activation function to produce the final result. The prediction results are maintained in their original format for loss calculation. During the inference phase, the outputs from all scales are concatenated and decoded into bounding boxes using an anchor generator and discrete distribution decoder, yielding the final detection results.

The prediction process of the LDHead module can be mathematically formalized as follows: Let the input feature be $x_{i}\in R^{C_{i}\times H_{i}\times W_{i}}$, which is first processed through channel adjustment and shared convolution to obtain a unified feature $F_{i}$. The prediction output for each position is:

(12) $$y_{i}=\mathrm{Concat}\;\left(\mathrm{Scale}\left(W_{\mathrm{reg}}*F_{i}\right),\sigma \left(W_{\mathrm{cls}}*F_{i}\right)\right)$$

Here, $W_{\mathrm{reg}}$ and $W_{\mathrm{cls}}$ represent the convolutional weights for the regression and classification branches, respectively, while σ(·) denotes the Sigmoid activation function.

The regression branch outputs a discrete probability distribution over bounding-box offsets; this distribution is then decoded—under Distribution Focal Loss (DFL) supervision—into continuous bounding-box parameters:

(13) $${\hat{b}}_{i}=\mathrm{dist}2\mathrm{bbox}\;\left(\Gamma_{I}\left(y_{i,\mathrm{reg}}\right),\mathrm{anchors}\right)$$

The final output is generated by concatenating the bounding box coordinates ${\hat{b}}_{i}$ and the category probability $y_{i,\mathrm{cls}}$ into a unified prediction tensor. During the inference stage, these are merged to generate the final detection results.

The LDHead module demonstrates three core advantages: lightweight design, high stability, and robust performance. First, its cross-scale shared convolution significantly reduces parameter count and computational load, enabling faster inference speeds than traditional independent detection heads—a critical requirement for real-time automotive systems. Second, the adoption of GroupNorm instead of BatchNorm effectively mitigates distribution drift during small-batch training or dynamic scenarios, ensuring feature consistency and generalization under unstable image statistics in adverse weather conditions. Additionally, the discrete distribution-based regression strategy enhances bounding-box prediction accuracy, allowing the model to reliably recover target positions even with low-quality inputs. By integrating shared convolution, depthwise separable architecture, and stable normalization, LDHead achieves both lightweight design and computational efficiency. Together with CE and AENet, this module forms the core improvement framework of ECL-YOLOv11: CE enhances edge feature expression, AENet optimizes multi-scale semantic fusion, while LDHead strikes a balance between efficient prediction and real-time deployment.

### 3.4. Module Integration and Overall Architecture

The ECL-YOLOv11 architecture is designed to enhance model robustness and detection accuracy in adverse weather conditions while maintaining the efficiency of the YOLO series. Compared with YOLOv11, ECL-YOLOv11 achieves end-to-end optimization—from feature extraction to detection—through three complementary modules: the CE module focuses on edge enhancement, the AENet module enables multi-scale contextual fusion, and the LDHead module ensures lightweight, efficient inference.

**Figure 9:**
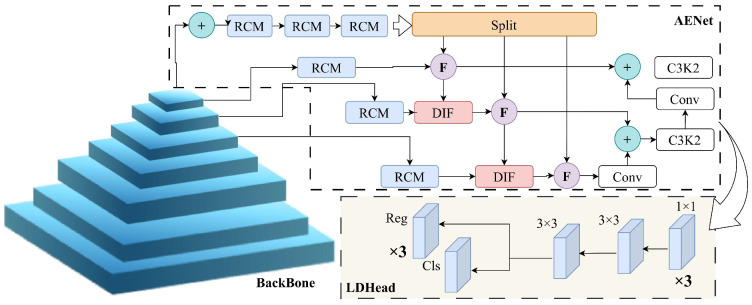
Architecture diagram of ECL-YOLOv11, Notes: CE: Convolutional Edge-Enhancement module; AENet: Context-Guided Multi-Scale Fusion Network; LDHead: Lightweight Shared Convolutional Detection Head; RCM: Rectangular Calibration Module; C3K2: Cross-Stage Partial Connection block; DIF: Down-to-Up Information Flow module.

As shown in Figure 9, the three components are sequentially integrated across different network layers to form a closed-loop feature flow characterized by “edge preservation, semantic fusion, efficient prediction.” At the Backbone stage, the CE module explicitly incorporates Sobel edge features, enhancing low-level structural information and providing high-quality texture input for subsequent fusion. In the Neck stage, AENet achieves cross-scale semantic alignment through pyramid context extraction and bidirectional feature flow, enabling consistent perception of small targets and distant objects. At the Head stage, LDHead employs shared convolutions and GroupNorm to achieve lightweight inference and stable normalization, maintaining detection accuracy and real-time performance under adverse weather conditions. This modular integration design establishes a collaborative mechanism from low-level structure enhancement to high-level prediction optimization, effectively addressing three major challenges faced by traditional detectors in low-visibility environments: edge degradation, scale mismatch, and inference latency.

## 4. Experiments

### 4.1. Dataset

**Figure 10:**
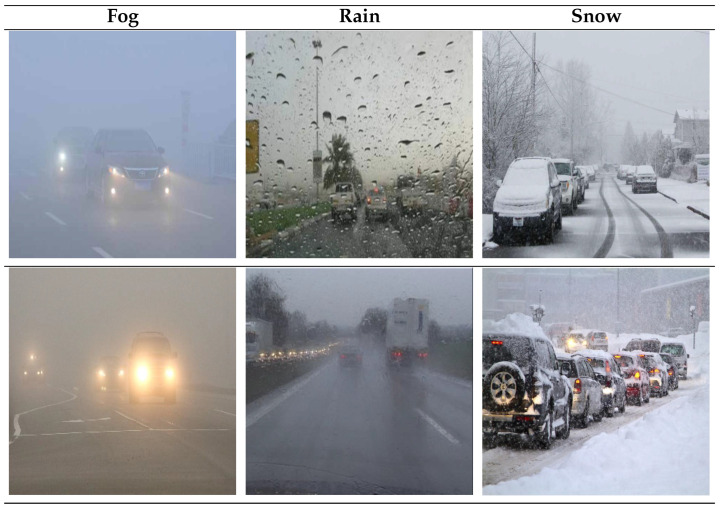
Sample images from the dataset.

**Figure 11:**
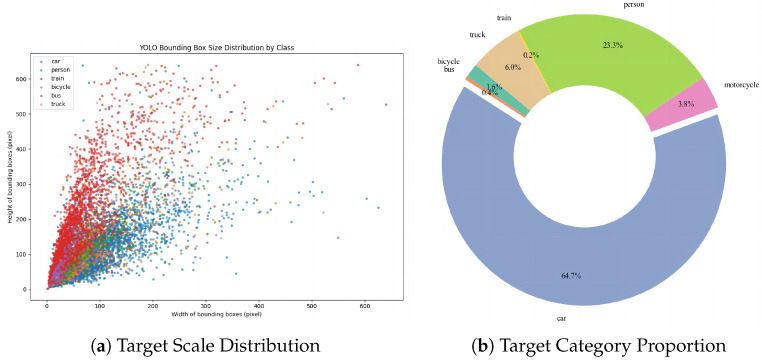
Distribution of dataset targets.

This study utilizes a self-developed dataset of severe weather traffic scenarios, comprising 9006 road traffic images captured under three typical meteorological conditions: rain, fog, and snow. The images were acquired using vehicle-mounted high-definition cameras, covering urban roads and highways with varying lighting conditions, visibility levels, and complex backgrounds. The dataset encompasses seven categories of traffic participants, including bicycles, buses, cars, pedestrians, trains, and trucks. All images are annotated in YOLOv11 format to ensure data consistency and effective model training. To guarantee reproducibility and standardized data utilization, the dataset is managed and exported through the Roboflow platform. The dataset is divided into an 8:1:1 ratio (80% for training, 10% for validation, and 10% for testing) to ensure fair and statistically reliable model performance evaluation. Selected images from the dataset are shown in Figure 10, while the data distribution is illustrated in Figure 11.

### 4.2. Experimental Setup and Training

The experimental setup is detailed in Table 1. To prevent overfitting on a relatively small dataset, we incorporate an Early Stopping mechanism and monitor validation performance throughout training. Training is capped at 600 epochs and terminates early if the validation mAP does not improve for 20 consecutive epochs. In addition, we adopt Cosine Annealing learning-rate decay and Label Smoothing to enhance model stability and generalization.

**Figure 12:**
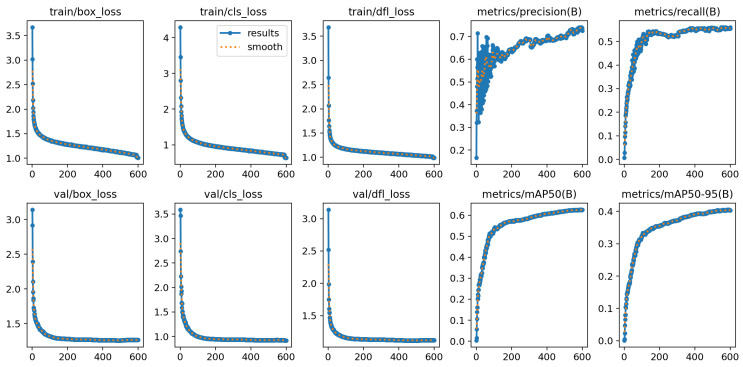
Training performance metrics.

**Figure 13:**
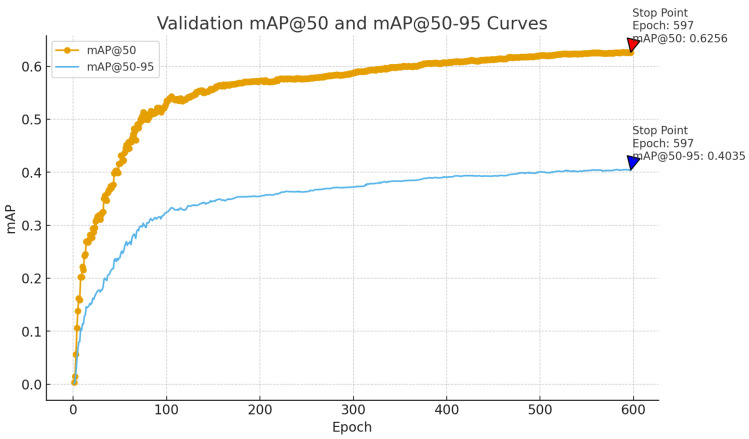
mAP convergence curve of the validation set.

As shown in Figure 12, the experimental results demonstrate the model’s convergence and stability during both training and validation phases. The loss functions (box_loss, cls_loss, and dfl_loss) exhibit a clear pattern of rapid decline followed by gradual stabilization as training progresses. The consistency between the validation and training curves indicates stable and synchronized convergence behavior. Notably, precision and recall rates initially increase before stabilizing, reflecting the model’s balanced accuracy–recall performance. To further validate the model’s convergence characteristics and generalization capabilities, this study adds the mAP convergence curve for the validation set to the existing training curve analysis (as shown in Figure 13).

Figure 13 illustrates the evolution of mAP@50 and mAP@50–95 during validation: both curves show sustained growth before saturating, with values stabilizing at 0.6256 and 0.4035, respectively, at epoch 597. This progression aligns with the loss trends observed during training, further confirming stable convergence and strong generalization across multi-scale and complex scenarios. The labeled Stop Point indicates that the Early Stopping mechanism terminated training once validation performance had saturated, avoiding unnecessary additional fitting. Moreover, both core metrics: mAP@50 and mAP@50–95, rise steadily throughout training before saturating, demonstrating robust detection performance and sustained learning capacity in diverse, challenging environments.

The experimental results collectively show that the proposed ECL–YOLOv11 achieves stable convergence during training, demonstrates stable convergence and generalization during training, and exhibits strong generalization. This experimental setup and training strategy provide reliable support for subsequent performance analysis and model validation under complex meteorological conditions.

### 4.3. Detection Performance Evaluation

To comprehensively validate the effectiveness of the ECL-YOLOv11 model for vehicle-mounted object detection, we evaluate three key metrics: mAP (mean Average Precision), Precision, and Recall. Together, these metrics assess performance from three perspectives—classification accuracy, bounding-box localization, and overall robustness

**Figure 14:**
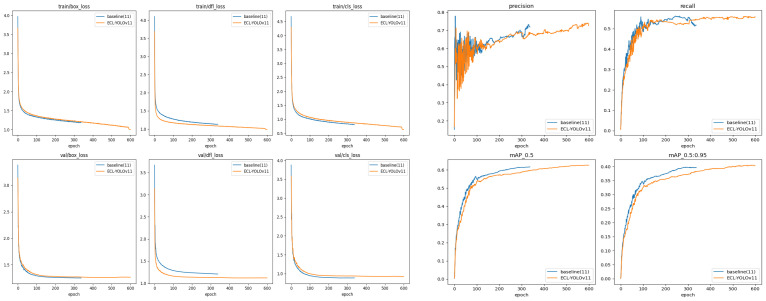
Comparison experimental results between ECL-YOLOv11 and YOLOv11.

The comparison between the baseline YOLOv11 and ECL-YOLOv11 is reported in Table 2 and Figure 14. ECL-YOLOv11 attains 62.7% mAP@50 and 40.5% mAP@50–95, which respectively measure average detection accuracy under single- and multi-threshold settings and reflect both classification and localization capability. Compared with YOLOv11 (61.4% and 39.7%), the gains of 1.3% and 0.8% indicate improved robustness and generalization, with more stable behavior in complex weather. In terms of Precision, ECL-YOLOv11 reaches 73.1% (vs. 68.8% for YOLOv11, +4.3%), demonstrating effective reduction of false positives and class confusion under edge blurring, uneven illumination, and rain/fog occlusion. For Recall, ECL-YOLOv11 achieves 55.6% (vs. 55.3%), indicating that the model maintains strong recall while improving detection accuracy. These results suggest that the proposed multi-module collaborative strategy achieves a better balance between sensitivity and recall without sacrificing either metric. Overall, ECL-YOLOv11 outperforms the baseline under harsh weather, with particularly meaningful improvements in Precision and mAP. Moreover, as shown in the Figure 14, the model exhibits no overfitting during training, and the observed gains stem from architectural improvements rather than tuning bias.

Overall, ECL-YOLOv11 not only improves the overall detection accuracy, but also achieves a favorable balance between performance and efficiency without increasing computational complexity, thereby providing a more practically deployable detection solution for in-vehicle vision systems.

### 4.4. Ablation Study

**Figure 15:**
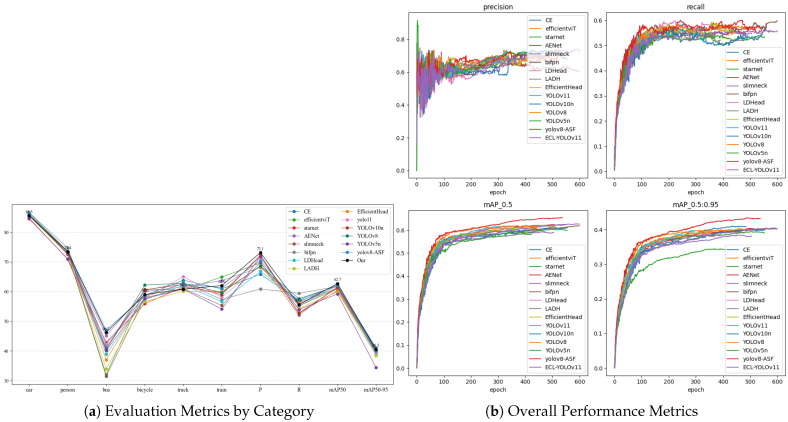
Ablation study evaluation metrics.

To visually demonstrate the effectiveness of the improved modules and the overall performance gains, ablation experiments were conducted. The primary objective is to progressively remove and combine different modules (CE, AENet, and LDHead), emphasizing the individual effectiveness of each module and the overall performance benefits of the final solution. The experimental results are presented in Table 3 and Figure 15.

As shown by the ablation results in Table 3, an overall mAP improvement of +1.3% was consistently observed across multiple independent experiments, demonstrating the stability and reproducibility of the proposed module. Given prior evidence that YOLOv11 performance approaches saturation under clear-weather conditions, the stable gains achieved under adverse weather are therefore particularly significant.

As shown in Table 3 and Figure 15, the baseline model (YOLOv11) achieved mAP@50 and mAP@50–95 of 61.4% and 39.7%, respectively, without structural augmentation. When the CE module was introduced, mAP@50 increased to 62.0% and mAP@50–95 to 41.0%, indicating that edge-enhanced convolution (CE) effectively preserves texture details and improves boundary localization accuracy. Although the CE module introduces only a marginal increase in computational complexity (GFLOPs 6.3 → 6.4), its stable performance under adverse weather suggests meaningful compensation for weather-induced structural degradation. Introducing AENet further strengthens feature fusion, achieving mAP@50 of 62.3% and mAP@50–95 of 40.4%, demonstrating that multi-scale semantic integration effectively improves overall detection accuracy. However, Precision decreases slightly from 68.8% to 68.1%, implying that the more complex fusion adopts a more conservative decision boundary to reduce false positives, potentially sacrificing recall. These results indicate that AENet excels at improving detection stability and identifying mid- to long-range blurred targets, while showing minor fluctuations in pure precision; overall, AENet balances feature expressiveness and robustness. The LDHead module introduces a decoupling mechanism in the detection head, enabling more independent classification and regression and thereby enhancing modeling capacity; without backbone augmentation, it attains mAP@50 of 60.9% and mAP@50–95 of 39.0%, slightly lower but lighter for deployment. With GFLOPs of just 5.6 and FPS reaching 451.6, it verifies the clear efficiency advantages of the shared-convolution mechanism for real-time detection.

Furthermore, at the module combination level, the CE+AENet architecture achieves 62.7% mAP@50 and 40.2% mAP@50–95 precision, demonstrating complementary edge enhancement and multi-scale context fusion. However, its inference speed drops to 215.3 FPS, indicating increased computational overhead. When LDHead is added to form the CE+AENet+LDHead structure, mAP@50 remains at 62.7%, mAP@50–95 slightly improves to 40.5%, while FPS significantly increases to 237.8 and GFLOPs decrease to 7.5. This “precision-preserving acceleration” phenomenon shows that LDHead optimizes computational paths and resource allocation through parameter sharing while maintaining detection accuracy, achieving structural-level performance balance. Category-wise analysis reveals that ECL-YOLOv11 demonstrates significant improvements over the baseline for medium-large targets (e.g., buses and trains), while maintaining stable performance for small targets such as pedestrians and bicycles, further evidencing AENet’s effective context fusion across different object scales. Precision increases to 73.1%, a 4.3% improvement over the 68.8% baseline, indicating advantages in reducing false positives and edge confusion.

In summary, the ablation experiments quantitatively demonstrate the structural design advantages of the AENet, LDHead, and CE modules proposed in this paper. These experiments validate that the improved ECL-YOLOv11 network not only achieves the synergy and complementary strengths of the modules but also effectively reduces model complexity and enhances inference speed while improving object detection accuracy compared to the baseline model, YOLOv11. The system demonstrates a balanced capability of maintaining high accuracy while ensuring real-time performance and lightweight design, providing a solid structural foundation and performance guarantee for a vehicle-based target detection system in adverse weather conditions.

## 5. Comparison Experiments

### 5.1. Comparison of Different Modules and Models

**Figure 16:**
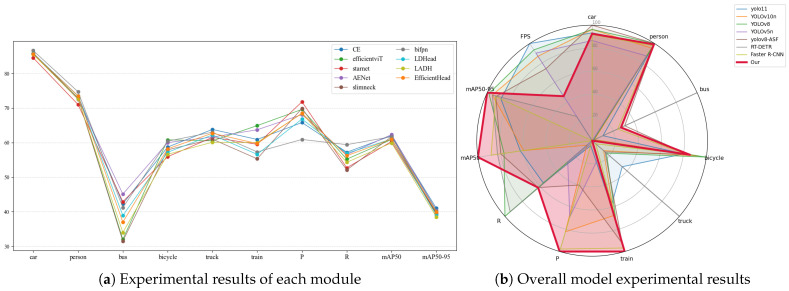
Overall performance metrics of the comparative experiment.

In the comparison experiments of this section, this paper conducts tests from four aspects (namely Backbone, Head, Neck, and overall model) to demonstrate the impact of different architectures or modules on the performance of the YOLOv11 model, as shown in Table 4 and Table 5 and Figure 16.

The comparison experiment data in Table 4 and Table 5 clearly shows that the improved model, ECL-YOLOv11, exhibits significant superiority in the object detection task.

The comparative results at the backbone level demonstrate that the proposed Edge-Enhanced Convolutional Module (CE) achieves superior performance in object detection. CE outperforms EfficientViT [22] (60.9%/39.7%) and StarNet [23] (60.3%/39.4%) with 62.0% mAP@50 and 41.0% mAP@50–95. Notably, while StarNet attains 71.8% Precision, its Recall drops to 52.7%, revealing limitations in modeling distant and small targets under complex weather conditions. By explicitly enhancing edge and texture cues, CE effectively mitigates feature degradation in hazy weather, thereby exhibiting stronger stability and robustness across multi-scale scenarios.

In the Neck architecture, AENet achieves an mAP@50–95 of 40.4%, surpassing SlimNeck [24] (38.4%) and BiFPN [25] (40.3%), and showing superior feature-fusion consistency in complex weather. Although BiFPN reaches 59.4% Recall, its limited mAP gain indicates insufficient stability of the weighted-fusion strategy under noisy conditions. Leveraging context-guided multi-scale semantic fusion, AENet strengthens spatial hierarchy modeling and markedly improves small-object recognition in low-contrast, long-range scenarios, demonstrating enhanced feature robustness in fog, rain, and snow.

In the comparison of detection head architectures, LDHead demonstrated competitive overall performance with an mAP@50–95 of 39.0%, comparable to EfficientHead (39.9%) and higher than LADH (38.5%). While EfficientHead showed higher Precision and Recall, its confidence fluctuations were more pronounced in complex backgrounds. LDHead, however, enhanced the independence between classification and regression through shared convolution and a decoupled design, reducing multi-task interference and yielding more stable predictions—particularly in scenes with edge blurring and small targets.

**Figure 17:**
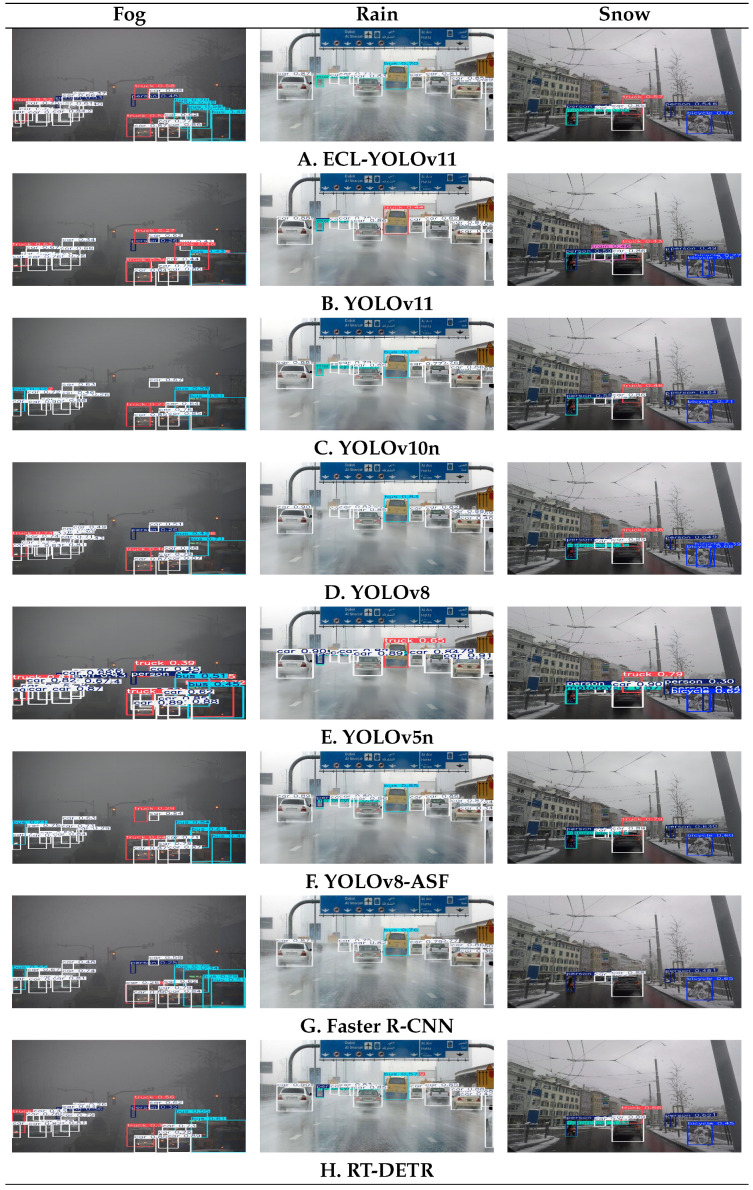
Qualitative visualization results of different models.

The comparative analysis shows that, in our experiments, ECL-YOLOv11 achieves 62.7% mAP@50 and 40.5% mAP@50–95 with 73.1% Precision and 55.6% Recall, outperforming the compared YOLO-series baselines—YOLOv11 (61.4%/39.7%), YOLOv10n [26] (61.3%/40.2%), and YOLOv8 [27] (61.9%/40.4%). Compared with non-YOLO detectors such as RT-DETR and Faster R-CNN, ECL-YOLOv11 attains comparable accuracy (62.1% vs. 62.3%) while delivering significantly faster inference, highlighting robustness under low-visibility, high-interference conditions. These results further validate the synergistic advantages of the ECL design in backbone feature extraction, context-guided fusion, and decoupled detection, achieving a balanced trade-off between accuracy and real-time efficiency with moderate complexity and strong deployment potential. Visual comparison results are presented in Figure 17.

As shown in Figure 17, systematic comparative analysis of eight models under extreme weather conditions clearly demonstrates ECL-YOLOv11’s significant advantages in detection accuracy, stability, and robustness. Foggy environments pose one of the most challenging scenarios for object detection, where extremely low visibility often leads to insufficient box confidence and missed small targets. Models like YOLOv11, YOLOv5n [28], and YOLOv8 generally produce numerous low-confidence boxes (0.2–0.4) in such conditions, exhibiting instability in detecting key targets like people and buses, with both false positives and missed detections. While YOLOv10n and YOLOv8-ASF show slight improvements in vehicle detection, their performance remains inconsistent in identifying trucks and people. RT-DETR achieves relatively accurate vehicle detection through its global attention mechanism, but maintains low confidence levels (0.3–0.6) and struggles with small-object recognition. Faster R-CNN performs the weakest in this scenario, only detecting nearby vehicles while failing to identify distant or small targets. In contrast, ECL-YOLOv11 demonstrates superior detection consistency, maintaining over 50% confidence for most targets and achieving relatively accurate recognition of buses and trucks, with overall stability clearly surpassing the other models. This indicates that its feature-extraction and multi-scale information-fusion strategy offers stronger adaptability to low-contrast targets.

In rainy scenes, light reflections and water-mist interference impose higher demands on detection models. Comparative results show that YOLOv11 misidentifies buses as trucks with blurred category boundaries, indicating insufficient generalization in complex environments; YOLOv5n exhibits similar errors, further revealing its limitations in cross-category recognition. While YOLOv8 and YOLOv8-ASF maintain higher confidence for bus detection (≈0.77–0.85) than YOLOv5n and YOLOv11, they still show confidence fluctuations for individual vehicles. RT-DETR detects vehicles effectively in rain but suffers a notable confidence drop for buses (≈0.57) due to strong reflections, suggesting that its global-attention mechanism is susceptible to focus drift under complex lighting. Although Faster R-CNN produces relatively accurate bounding boxes, its overall confidence remains low and it fails to stably detect distant small objects. By contrast, ECL-YOLOv11 maintains stable bus recognition and exhibits consistent confidence across different vehicle classes over multiple lanes without misclassification. This stability is crucial for rainy-day traffic monitoring and indicates stronger discrimination under reflection and occlusion interference.

In snow scenes, background noise, occlusion, and surface coverage in snow substantially affect detection accuracy. YOLOv11 yields false positives for “train,” reflecting insufficient discriminative robustness in challenging environments. Although YOLOv5n achieves high confidence for trucks, its confidence for person remains around 0.30–0.34, implying a serious missed-detection risk. YOLOv10n and YOLOv8 perform relatively stably but still struggle with low truck confidence or insufficient bicycle detection; YOLOv8-ASF shows advantages for motorcycles and bicycles, yet some boxes retain low confidence. Faster R-CNN fails to detect primary targets effectively, providing only low-confidence boxes at close range, whereas RT-DETR detects trucks (≈0.66) but fails on pedestrians and non-motorized classes—indicating an attention bias of Transformer architectures under high-illumination scenes. In contrast, ECL-YOLOv11 maintains stable multi-class recognition in snow, accurately localizing trucks, cars, persons, and bicycles with a reasonable confidence distribution. Unlike YOLOv11’s misclassifications or YOLOv5n’s extremely low confidences, ECL-YOLOv11 shows stronger robustness under complex backgrounds and noise interference.

Overall. ECL-YOLOv11 delivers consistent improvements over mainstream models and structural alternatives, achieving effective synergy in feature retention, semantic fusion, and lightweight detection. The results indicate modest yet stable metric gains accompanied by consistent, repeatable behavior in complex real-world conditions, underscoring the framework’s scientific soundness and engineering applicability. This provides a solid foundation—and a scalable pathway—for future multimodal fusion and real-time perception in autonomous-driving environments.

### 5.2. Comparison Experiments Considering Adverse Weather Conditions and Relative Motion States

**Figure 18:**
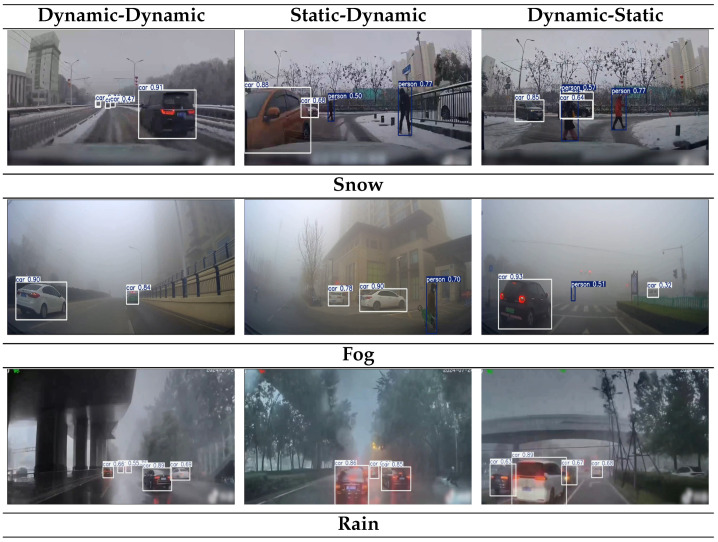
Model validation results considering adverse weather conditions and motion states.

To further verify the effectiveness of ECL-YOLOv11 as an in-vehicle visual object detection solution under adverse weather conditions, experiments were designed in three weather environments: snow (Snow), fog (Fog), and rain (Rain). The model’s performance was tested in three relative motion state scenarios: dynamic (autonomous vehicle)–dynamic (detected target), static (autonomous vehicle)–dynamic (detected target), and dynamic (autonomous vehicle)–static (detected target). As shown in Figure 18, the experiment primarily examines the robustness of ECL-YOLOv11 in small target detection, long-distance target localization, and complex background interference.

As shown in Figure 18, the experiment configured three relative-motion combinations under each weather condition to simulate typical visual-perception tasks for autonomous driving in complex environments. Key evaluation priorities included small-object detection performance, long-distance target localization accuracy, and false-detection control in cluttered backgrounds. In foggy scenarios, ECL-YOLOv11 accurately detected nearby vehicles in both dynamic–dynamic and dynamic–static states, maintaining detection confidences of 0.84–0.93; it also kept confidences above 0.50 for distant vehicles and pedestrians under severely limited visibility, markedly reducing missed-detection risk due to blur and occlusion, thereby validating the CE module’s effectiveness in restoring structural details and contour recognition. Under rainy conditions, the model maintained robust performance in dynamic–dynamic and static–dynamic settings: despite distortions from raindrops, haze, and roadway reflections, major-vehicle confidences stayed above 0.85, boxes were complete, and long-range localization remained stable, indicating that the AENet module, via context-guided multi-scale fusion, improved semantic consistency in low-contrast dynamic scenes. Snow experiments further verified generalization in complex backgrounds and multi-target mixed scenes: vehicle confidences remained 0.85–0.91 in static→moving and moving→static states, while pedestrian detection stabilized at 0.50–0.77; even with snow glare and partial occlusion, ECL-YOLOv11 showed no notable class confusion or missed detections. Additionally, the lightweight LDHead ensured synchronization between inference speed and accuracy in static→moving and moving→static scenarios, demonstrating excellent latency control and structural adaptability. Overall, ECL-YOLOv11 exhibited stable detection across adverse weather and motion states, maintaining high confidences and complete detections under extreme conditions (insufficient illumination, low contrast, occlusion, reflection), and significantly mitigating boundary ambiguity and misidentification issues common to traditional detectors. Compared with conventional models, often limited by confidence fluctuation, labeling errors, and weaker environmental adaptability, our framework provides more robust, reliable target perception, offering strong support for intelligent driving in complex traffic environments.

## 6. Conclusions

This study presents ECL-YOLOv11, an edge-enhanced, context-guided, and lightweight object detection framework designed for vehicle-mounted visual perception under adverse weather conditions. Building upon the YOLOv11 baseline, the proposed model integrates three complementary modules:

(1) The Edge-Enhancement Convolution (CE) module, which explicitly preserves edge and gradient features to mitigate boundary degradation under low visibility;

(2) The Context-Guided Multi-Scale Fusion Network (AENet), which strengthens semantic consistency and multi-scale representation through context-aware feature aggregation;

(3) The Lightweight Shared-Convolution Detection Head (LDHead), which optimizes computational paths for real-time inference without sacrificing accuracy.

Comprehensive experiments demonstrate that ECL-YOLOv11 achieves modest yet stable and reproducible performance improvements in detection accuracy, robustness, and computational efficiency compared with mainstream detectors (YOLOv11, YOLOv8, YOLOv10n, RT-DETR, Faster R-CNN). Specifically, the model reaches mAP@50 = 62.7%, mAP@50–95 = 40.5%, and 237.8 FPS. Although the overall improvement (1.3 mAP) is numerically limited, it is consistently reproduced across multiple independently trained variants (as shown in Table 3), indicating that the observed gain is systematic rather than random variation. Under fog, rain, and snow, the framework maintains consistent detection confidence and avoids misclassification or missed targets common to traditional detectors, validating its robust adaptability across complex environmental and motion states. The design philosophy of ECL-YOLOv11 emphasizes a balance between structural interpretability and operational practicality. The synergy among the CE, AENet, and LDHead modules enhances both feature quality and system responsiveness, offering a solid foundation for robust in-vehicle perception.

Nevertheless, several limitations remain. The dataset used in this study (∼9 k images) is relatively small and focuses on adverse-weather scenarios; systematic evaluations under clear weather and larger-scale data remain to be performed. Additionally, the training process was constrained by computational resources (single GPU, 640 × 640 input resolution, batch size 16), which may limit the achievable upper bound of model performance; therefore, the improvements observed in this study should be interpreted as conservative estimates under current conditions. The model also relies solely on visual input, without leveraging complementary modalities.

Future research will therefore focus on three directions:

(1) Multi-sensor fusion, integrating ECL-YOLOv11 with LiDAR and millimeter-wave radar to improve depth perception and robustness in low-visibility environments;

(2) System-level co-optimization, coupling detection, planning, and decision-making modules in autonomous-driving pipelines to enable end-to-end collaborative learning;

(3) Dynamic-scene adaptation, developing online and continual-learning mechanisms to sustain long-term stability under evolving real-world conditions.

In summary, ECL-YOLOv11 demonstrates scientifically sound, repeatable, and practically deployable improvements in object detection under complex weather. By balancing robustness and real-time efficiency, it offers a feasible path toward reliable and efficient perception for intelligent vehicles, bridging the gap between algorithmic robustness and real-time application, and laying the groundwork for future multimodal and adaptive perception systems.

## Figures and Tables

**Table 1:** Experimental environment parameters.

Configuration	Detailed Description
CPU	Intel Core i5-12400F
GPU	NVIDIA GeForce RTX 3060 (12 GB VRAM)
Operating System	Windows 11
Software Environment	Python 3.10.15, PyTorch 2.5.0, CUDA 12.1
Framework Version	Ultralytics 8.3.9
Image Size	640 × 640
Training Period	600
Batch Size	16

**Table 2:** Experimental results metrics.

Model	P	R	mAP@50	mAP@50–95
YOLOv11	0.688	0.553	0.614	0.397
ECL-YOLOv11	0.731	0.556	0.625	0.405

**Table 3:** Evaluation metrics.

(a) Category-wise Evaluation Metrics.							
**Model**		**Car**	**Person**	**Bus**	**Bicycle**	**Truck**	**Train**
Baseline(11)		85.6	73.4	42.4	57.8	60.5	60.9
CE		85.8	73.5	42.3	58.7	63.8	60.9
AENet		85.7	72.5	45.1	60.0	63.6	63.7
LDHead		85.7	73.5	38.9	57.2	62.2	56.9
CE + AENet		85.3	72.6	42.8	59.9	62.0	60.5
AENet + LDHead		85.1	72.7	42.1	58.6	60.4	57.9
CE + LDHead		85.8	73.0	36.2	56.2	60.2	59.5
Our		85.5	73.4	46.2	59.0	60.8	62.0
(b) Overall Performance Metrics.							
**Model**	**P**	**R**	**mAP50**	**mAP50–95**	**Para.**	**GFLOPs**	**FPS**
Baseline(11)	68.8	55.3	61.4	39.7	2,583,517	6.3	406.5
CE	65.8	57.2	62.0	41.0	2,530,501	6.4	337.7
AENet	68.1	56.5	62.3	40.4	3,294,861	8.6	249.2
LDHead	66.8	56.9	60.9	39.0	2,420,882	5.6	451.6
CE + AENet	69.8	55.6	62.7	40.2	3,241,845	8.7	213.5
AENet + LDHead	67.0	57.1	61.1	39.9	2,934,413	7.2	142.5
CE + LDHead	65.4	57.2	60.5	39.2	2,263,029	5.1	209.9
Our	73.1	55.6	62.7	40.5	3,001,194	7.5	237.5

Note: GFLOPs: Billion Floating-Point Operations, used to measure the computational complexity of the model.

**Table 4:** Compare the test results of each module.

	Model	Car	Person	Bus	Bicycle	Truck	Train	P	R	mAP50	mAP50-95
Backbone	CE	85.8	73.5	42.3	58.7	63.8	60.9	65.8	57.2	62	41
	efficientViT	85.6	72.9	32.0	60.8	60.5	64.9	69.6	55.2	60.9	39.7
	starnet	84.5	71.0	42.9	55.9	61.5	59.5	71.8	52.7	60.3	39.4
Neck	AENet	85.7	72.5	45.1	60.0	61.6	63.7	68.1	56.5	62.3	40.4
	slimneck	85.9	72.8	31.5	57.9	61.0	55.3	69.9	52.1	59.1	38.4
	bifpn	86.6	74.7	41.2	60.4	63.0	57.3	69.0	59.4	61.7	40.3
Head	LDHead	85.7	73.5	38.9	57.2	62.2	56.9	66.8	56.9	61.0	39.0
	LADH	85.5	72.6	34.0	56.6	60.1	60.0	68.4	54.3	59.9	38.5
	EfficientHead	85.6	73.5	37.0	58.2	62.7	59.5	68.6	56.2	61.0	39.9

**Table 5:** Comparison of detection results of all holistic models.

Model	Car	Person	Bus	Bicycle	Truck	Train	P	R	mAP50	mAP50-95	FPS
yolo11	85.6	73.4	42.4	57.8	65.0	58.1	68.8	55.3	61.4	39.7	434.4
YOLOv10n	86.0	72.6	40.6	60.6	62.4	58.9	72.3	52.8	61.3	40.2	385.0
YOLOv8	86.0	73.6	40.4	62.2	62.8	59.6	68.6	57.6	61.9	40.4	409.7
YOLOv5n	84.7	70.9	40.1	57.6	60.8	54.1	71.7	53.9	59.2	39.4	397.2
yolov8-ASF	86.5	73.6	47.0	58.6	62.6	61.3	70.4	55.6	62.0	40.5	336.7
RT-DETR	73.0	52.5	62.3	40.0	73.0	52.5	68.7	57.2	62.1	40.2	152.0
Faster R-CNN	85.4	73.1	45.5	58.3	61.3	61.7	73.0	52.5	62.3	40.0	58.0
Our	85.5	73.4	46.2	59.0	60.8	62.0	73.1	55.6	62.7	40.5	230.3

## Data Availability

The data that support the findings of this study are available from the corresponding author upon reasonable request.
